# Assessment of Accuracy and Usability of a Fee Estimator for Ambulatory Care in an Integrated Health Care Delivery Network

**DOI:** 10.1001/jamanetworkopen.2019.17445

**Published:** 2019-12-13

**Authors:** Cheryl D. Stults, Jiang Li, Dominick L. Frosch, Hari Krishnan, Gregg Smith-McCurdy, Veena G. Jones, Albert S. Chan

**Affiliations:** 1 Sutter Health Center for Health Systems Research, Palo Alto, California; 2 Palo Alto Medical Foundation Research Institute, Palo Alto, California; 3 Sutter Health Office of Patient Experience, Sacramento, California; 4 Hill Physicians Medical Group, San Ramon, California; 5 Stanford Center for Biomedical Informatics Research, Stanford, California

## Abstract

**Importance:**

Given increased enrollment in high-deductible health insurance plans and mandates from the Patient Protection and Affordable Care Act, individualized price transparency tools are needed.

**Objective:**

To assess accuracy and initial user experience of a cost estimation tool for ambulatory procedures delivered via an online patient portal and informed by real-time data feeds from third-party payers.

**Design, Setting, and Participants:**

This quality improvement study included patients aged 18 years and older at an integrated health care system in Northern California. Data from patients who used the cost estimator tool from August 21, 2018, to April 9, 2019, and who had matching explanation of benefits statements were used to assess accuracy of the tool. User experience was assessed with a brief survey completed online or via postal mail. Data were analyzed from April 15, 2019, to October 11, 2019.

**Main Outcomes and Measures:**

Tool accuracy and user experience and satisfaction.

**Results:**

As of April 30, 2019, 4610 estimates (3569 [77.4%] via internet; 1041 [22.6%] via telephone) were produced using the cost estimator tool. Among 342 individuals who had an estimate and a matching explanation of benefits statement, 287 estimates (83.9%) were accurate. All 342 individuals with an estimate and an explanation of benefits statement were invited to participate in a user survey, and 125 individuals completed the survey (36.5% response rate). Survey respondents included 92 (73.6%) women, 72 (57.6%) non-Hispanic white participants , 91 participants (72.8%) with a college degree or higher, and 55 participants (44.0%) with an income of $100 000 per year or higher. Mean (SD) age was 46.8 (13.1) years. Ninety-nine participants (79.2%) found the tool easy to use, 109 participants (87.2%) would use it again, and 100 participants (80.0%) would recommend it to others. Seven participants (5.6%) reported contacting a clinician about the estimate, and 12 participants (9.6%) changed their decision based on the estimate.

**Conclusions and Relevance:**

This quality improvement study is the first report of an online cost estimator in an integrated health care delivery network. The findings suggest that the tool, informed by real-time data feeds from third-party payers, was easy to use and provided accurate results. Increasing the number of searchable services and sharing best practices with other health care systems who share the same portal platform are the next steps for the tool.

## Key Points

**Question:**

Can a cost estimation tool provide accurate, user-friendly, personalized information for ambulatory procedures via an online patient portal?

**Findings:**

This quality improvement study included 4610 estimates during a 10-month period and found that the new tool showed an accuracy rate of 83.9%. A survey of a subset of users found that most respondents were satisfied with their experience using the tool and would recommend it to others.

**Meaning:**

These findings suggest that a cost estimation tool can be easy to use and provide accurate real-time estimates integrated into the online patient portal.

## Teaser

This quality improvement study examines the accuracy and user experience of a cost estimator tool delivered via an online patient portal and informed by real-time data from third-party payers.

## Introduction

Health care has been slow to embrace price transparency. The Patient Protection and Affordable Care Act mandates price transparency, and a new Centers for Medicare & Medicaid Services rule requires hospitals to publish their fee schedule on their websites.^1^ This rule has led to widespread comment that it fails to achieve the objective of genuine price transparency because of wide variation in patients’ yearly deductibles, coinsurance, and contracted rates with health insurers relative to list prices.^2^

Recent estimates indicate that 46% of individuals in the United States younger than 65 years are covered through high-deductible health plans.^3^ Continued growth of these plans underlines the importance of effective tools to enable patients to compare prices and quality of services from different health care networks. Tools are available from commercial companies (eg, FairHealth),^4^ public websites (eg, CMS Hospital Compare), and employers and health insurance plans,^5^ but overall uptake remains low.^6,7^ Barriers to use include lack of knowledge, poor user interfaces, and inaccurate estimations.^7^ Easy-to-find, user-friendly, personalized cost estimation tools that account for patient’s insurance coverage are essential to address unmet needs.

To our knowledge, this is the first study to report on the development, accuracy, and initial user experiences of a cost estimator tool for ambulatory procedures delivered via an online patient portal and informed by real-time data feeds from third-party payers.

## Methods

The Sutter Health institutional review board approved this study. Survey respondents provided written informed consent. A waiver of consent was obtained to use the data from cost estimator tool users who did not respond to the survey owing to the minimal risk of the study. This study is reported following the Standards for Quality Improvement Reporting Excellence (SQUIRE), American Association for Public Opinion Research (AAPOR), and Standards for Reporting Qualitative Research (SRQR) reporting guidelines.

Sutter Health is an integrated health care system in Northern California serving more than 3 million patients. An early adopter of patient-centered approaches to health care delivery, Sutter Health was the first health care system in the nation to implement MyChart, Epic Systems’ vendor-based patient portal, branded as *My Health Online* (MHO).^8,9^ As of 2018, approximately 79% of patients of Sutter Health ambulatory care are enrolled in MHO.^10^

Sutter Health created the Consumer Accessible Fee Estimation initiative to provide price transparency for patients to use of their own initiative. The initial cost estimator tool implementation includes 220 common services from Sutter Health’s top 10 insurance payers by volume. The patient can search for services by key words, by *Current Procedural Terminology* code, or by choosing from a list within categories. These categories include immunizations and vaccines, laboratory tests, heart or lung tests, office visits, specialist consultations, and imaging services (eg, magnetic resonance imaging, computed tomography scans, mammography, radiographic imaging, ultrasonographic imaging). When a patient initiates a query about a *Current Procedural Terminology* code or selects from an established estimate template, the service cost is calculated from the fee schedule in the electronic health record system. The contracted rate is calculated from historical data or contracts held by the system. Final patient responsibility is calculated leveraging service cost, contracted rate, and a personalized out-of-pocket cost calculation inclusive of copay, coinsurance, and deductibles, derived from a real-time query sent to the payer.

Development of this tool required a total of 7000 hours during an 18-month period by 7 full-time employees. The primary challenge in development was data mapping issues related to meeting the Electronic Data Interchange standards that are part of the Health Insurance Portability and Accountability Act. One part of the Electronic Data Interchange standards is the 270 and 271 transaction sets. The 270 Transaction Set transmits health care eligibility benefit inquiries from health care practitioners, insurers, and other health care adjudication processors, while the 271 Transaction Set is the response mechanism for these inquiries.^11^ In addition to current enrollment status, these transaction sets also contain fields that include deductibles, copays, and coinsurances. However, some of these values (eg, copays and deductibles) are service-dependent and can thus be applied to encounters differently based on payer, plan, visit type, or service type. An additional source of variation is that while Electronic Data Interchange standards do require discrete data fields within a defined record format, they do not mandate the standard values for those fields. For example, one organization may categorize a procedure as “radiology services” while a different receiving organization may classify that same procedure as “imaging services.” To ensure correct data mapping, Sutter Health information analysts needed to analyze each transaction set for all 220 services for all payers, plans, and benefits to determine various irregularities and to appropriately account for these within the cost estimator.

An additional challenge in the cost estimator tool development regards issues with data aggregation. Health care transactions are typically processed by a myriad of local, regional, and national clearinghouses. As transactions are transferred from vendor to vendor, they are repeatedly collected, reformatted, and transmitted, which increases costs, delays, and errors. Senior leaders at Sutter Health invested in building collaborative relationships and data sharing arrangements with industry partners. Sutter Health partnered with several third-party insurance payers and an eligibility vendor to facilitate the information exchange necessary to develop fee estimates. These partnerships enabled the flowing of data necessary to ensure accurate data mapping for the cost estimator tool.

The patient cost estimator tool launched on June 1, 2018. We present the overall number of estimates from initiation through April 30, 2019, and we present accuracy rates through April 9, 2019.

We designed a short, 16-question, self-administered patient survey to understand patient experience with the cost estimator for overall usability, satisfaction, loyalty, and suggestions for improvements. We did not pilot test the survey because several questions came from existing validated measures.^12,13,14^ Our nonprobability sample included a subset of users, including current patients of Sutter Health 18 years and older who had matching explanation of benefits (EOB) statements from August 21, 2018, to April 9, 2019. Our sample only included those with an EOB because we wanted to verify the accuracy of the tool estimates. Claims can be submitted to the payer for reimbursement only if the service is performed and completed. Once the claim is processed by the payer, an EOB is received and loaded into the Sutter Health electronic health records system to compare it with the estimate. We sent the study invitation and link to an online REDCap survey (Vanderbilt University) through MHO to eligible patients. After 2 weeks, we mailed nonrespondents a paper version of the survey. Respondents who completed the survey received a $20 gift card. Data collection occurred from April 16, 2019, to June 10, 2019.

### Statistical Analysis

Descriptive statistics are presented for survey data. Survey respondents reported their race/ethnicity to determine potential differences across groups and estimate accuracy. We defined *accuracy* as a difference between estimate and billed amount of less than $10 or 5%. Fisher exact test was used to compare categorical variables with 2-tailed α set at .05. Data were analyzed using SAS version 9.4 (SAS Institute). One of us (C.D.S), a qualitative sociologist, coded responses to open-ended questions into emergent thematic groupings to determine their frequency. Data analysis was conducted from April 15, 2019, to October 11, 2019.

## Results

As of April 30, 2019, 4610 total estimates were produced using the cost estimator tool, including 3569 estimates (77.4%) initiated online by patient self-service queries via MHO and 1041 estimates (22.6%) created after patient-initiated queries to the Sutter Health Patient Telephone Service Center.

Of 4610 total estimates, 342 individuals (7.5%) had a matching EOB. As of April 9, 2019, 33 individuals with an EOB (9.6%) had called the Sutter Health Patient Telephone Service Center with concerns regarding significant variance between the estimates from the cost estimator and the subsequent billing statement. The remaining 287 individuals (83.9%) had equal or less payer responsibility as that estimated by the cost estimator tool (Table 1). Among 342 individuals with estimates and EOBs, 239 (69.9%) were women and 223 (65.2%) were younger than 55 years. We invited all 342 patients meeting inclusion criteria to complete the survey and received 125 completed surveys (36.5% response rate). There were no statistically significant differences between respondents and nonrespondents. Respondents included 92 women (73.6%), 72 (57.6%) non-Hispanic white participants, 91 participants (72.8%) with a 4-year college degree or higher, and 55 participants (44.0%) reported an income of more than $100 000 per year (Table 2). Mean (SD) age was 46.8 (13.1) years (range, 24-79 years). One hundred fourteen respondents (91.2%) had some type of private preferred provider organization insurance coverage. The most frequently searched-for services were in the category of imaging services (85 searches [68.0%]) and laboratory testing (25 searches [20.0%]). There were no statistically significant differences regarding tool accuracy and sex, race/ethnicity, education level, income, age, insurance coverage, or searched services. Ninety-nine participants (79.2%) found the cost estimator tool easy or very easy to use, 109 participants (87.2%) would use the tool again, and 100 participants (80.0%) would recommend it to others. There were no statistically significant differences regarding tool accuracy and ratings for ease of use, use again, and recommend to others. Participants who had inaccurate estimates were more likely to feel neutral that the tool was helpful in planning for their health care needs than those who had accurate estimates (7 participants [31.8%] vs 10 participants [9.7%]; *P* = .007) and were less likely to receive services from Sutter Health (9 participants [40.9%] vs 72 participants [69.9%]; *P* = .004). Only 12 participants (9.6%) reported changing their decision based on tool use, with 4 participants (3.2%) deciding to have the service at Sutter Health. Similarly, 7 participants (5.6%) contacted their clinician about the estimate to discuss potential options and verify cost (Table 2).

**Table 1.** Cost Estimation Accuracy Rate Among Patients Who Used the Sutter Cost Estimator Tool With Matching EOB Statements

Category	No. (%)		
	Survey Respondent (n = 125)	Survey Nonrespondent (n = 217)	Total (N = 342)
Accurate or patient less than estimate^a^	103 (82.4)	184 (84.8)	287 (83.9)
Estimate matched with billed amount: difference between estimate and billed amount was ≤$10 or ≤5%	70 (56.0)	118 (54.4)	188 (55.0)
Patients had different or additional services at the time of visit owing to medical necessity; no payment by patient necessary	7 (5.6)	18 (8.3)	25 (7.3)
Estimated copay higher than copay in the EOB; no payment by patient necessary	0	2 (0.9)	2 (0.6)
Estimated deductible less than deductible in the EOB owing to timing issues; patient may have met their deductible after their estimate was created	22 (17.6)	33 (15.2)	55 (16.1)
Estimated coinsurance less than coinsurance in the EOB owing to timing issues; patient may have met their deductible after their estimate was created	4 (3.2)	13 (6.0)	17 (5.0)
Not accurate: patient paid more than estimate	22 (17.6)	33 (15.2)	55 (16.1)
Patients had different or additional services at the time of visit owing to medical necessity; payment by patient necessary	11 (8.8)	18 (8.3)	29 (8.5)
Estimated copay less than copay in the EOB; payment by patient necessary	2 (1.6)	2 (0.9)	4 (1.2)
Payer denied the claim owing to services not being covered or coding issues	1 (0.8)	0	1 (0.3)
Insurance changed from the time the estimate was created to when the service was provided	0	1 (0.5)	1 (0.3)
Estimated deductible less than deductible in the EOB; payment by patient necessary	4 (3.2)	9 (4.1)	13 (3.8)
Estimated coinsurance less than coinsurance in the EOB; payment by patient necessary	2 (1.6)	2 (0.9)	4 (1.2)
Contracted payers provided different contracted rates on the EOB compared with the estimate	2 (1.6)	1 (0.5)	3 (0.9)

Abbreviation: EOB, explanation of benefit.

^a^ Accuracy was defined as a difference between the estimate and billed amount of less than $10 or 5%.

**Table 2.** Survey Respondent Characteristics and User Experience

Characteristic	Participants, No. (%)			P Value
	Estimate Accurate^a^ (n = 103)	Estimate Not Accurate (n = 22)	Total (N = 125)	
Sex				
Women	77 (74.8)	15 (68.2)	92 (73.6)	.60
Men	26 (25.2)	7 (31.8)	33 (26.4)	
Age, y				
18-34	23 (22.3)	5 (22.7)	28 (22.4)	.66
35-44	24 (23.3)	7 (31.8)	31 (24.8)	
45-54	18 (17.5)	1 (4.5)	19 (15.2)	
55-64	31 (30.1)	8 (36.4)	39 (31.2)	
65-74	6 (5.8)	1 (4.5)	7 (5.6)	
≥75	1 (1.0)	0	1 (0.8)	
Race/ethnicity				
Missing	5 (4.9)	3 (13.6)	8 (6.4)	.14
American Indian or Alaska Native	0	1 (4.5)	1 (0.8)	
Hispanic	11 (10.7)	3 (13.6)	14 (11.2)	
Non-Hispanic Asian	20 (19.4)	5 (22.7)	25 (20.0)	
Non-Hispanic black	1 (1.0)	1 (4.5)	2 (1.6)	
Non-Hispanic Native Hawaiian or Pacific Islander	2 (1.9)	0	2 (1.6)	
Non-Hispanic white	63 (61.2)	9 (40.9)	72 (57.6)	
Other	1 (1.0)	0	1 (0.80)	
Education level				
High school graduate or GED	5 (4.9)	2 (9.1)	7 (5.6)	.38
Some college or 2-y college degree	24 (23.3)	3 (13.6)	27 (21.6)	
4-y college degree	36 (35.0)	11 (50.0)	47 (37.6)	
>4-y college degree	38 (36.9)	6 (27.3)	44 (35.2)	
Annual household income				
Missing	24 (23.3)	5 (22.7)	29 (23.2)	.14
>$200 000	15 (14.6)	0	15 (12.0)	
$150 001-200 000	12 (11.7)	5 (22.7)	17 (13.6)	
$100 001-150 000	17 (16.5)	6 (27.3)	23 (18.4)	
$50 001-100 000	26 (25.2)	5 (22.7)	31 (24.8)	
≤$50 000	9 (8.7)	1 (4.5)	10 (8.0)	
Insurance plan				
Private insurance preferred provider organization	94 (91.3)	20 (90.9)	114 (91.2)	.82
Private insurance health maintenance organization	5 (4.9)	2 (9.1)	7 (5.6)	
Public insurance	2 (1.9)	0	2 (1.6)	
Other	2 (1.9)	0	2 (1.6)	
Estimated service				
Imaging	69 (67.0)	16 (72.7)	85 (68.0)	>.99
Laboratory testing	21 (20.4)	4 (18.2)	25 (20.0)	
Immunizations	8 (7.8)	1 (4.5)	9 (7.2)	
Heart testing	4 (3.9)	1 (4.5)	5 (4.0)	
Specialist visit	1 (1.0)	0	1 (0.8)	
**User Experience**				
Using the cost estimator tool was				
Missing	5 (4.9)	0	5 (4.0)	.861
Difficult or very difficult	3 (2.9)	0	3 (2.4)	
Neutral	14 (13.6)	4 (18.2)	18 (14.4)	
Easy or very easy	81 (78.6)	18 (81.8)	99 (79.2)	
Satisfied with amount of time required				
Missing	1 (1.0)	0	1 (0.8)	>.99
Disagree	4 (3.9)	1 (4.5)	5 (4.0)	
Neutral	5 (4.9)	1 (4.5)	6 (4.8)	
Agree	93 (90.3)	20 (90.9)	113 (90.4)	
Satisfied with experience				
Missing	1 (1.0)	0	1 (0.8)	.58
Disagree	13 (12.6)	1 (4.5)	14 (11.2)	
Neutral	6 (5.8)	2 (9.1)	8 (6.4)	
Agree	83 (80.6)	19 (86.4)	102 (81.6)	
Helpful to plan for needs				
Missing	2 (1.9)	0	2 (1.6)	.007
Disagree	17 (16.5)	0	17 (13.6)	
Neutral	10 (9.7)	7 (31.8)	17 (13.6)	
Agree	74 (71.8)	15 (68.2)	89 (71.2)	
Improves overall experience at Sutter Health				
Missing	1 (1.0)	0	1 (0.8)	>.99
Disagree	9 (8.7)	2 (9.1)	11 (8.8)	
Neutral	14 (13.6)	3 (13.6)	17 (13.6)	
Agree	79 (76.7)	17 (77.3)	96 (76.8)	
Received services from Sutter Health				
Disagree	11 (10.7)	1 (4.5)	12 (9.6)	.004
Neutral	20 (19.4)	12 (54.5)	32 (25.6)	
Agree	72 (69.9)	9 (40.9)	81 (64.8)	
Would use again				
Missing	1 (1.0)	0	1 (0.8)	>.99
Disagree	4 (3.9)	1 (4.5)	5 (4.0)	
Neutral	8 (7.8)	2 (9.1)	10 (8.0)	
Agree	90 (87.4)	19 (86.4)	109 (87.2)	
Would recommend tool				
Missing	3 (2.9)	1 (4.5)	4 (3.2)	.56
Disagree	8 (7.8)	2 (9.1)	10 (8.0)	
Neutral	8 (7.8)	3 (13.6)	11 (8.8)	
Agree	84 (81.6)	16 (72.7)	100 (80.0)	
Changed decision				
No	81 (78.6)	20 (90.9)	101 (80.8)	.28
Yes	10 (9.7)	2 (9.1)	12 (9.6)	
I don’t know yet	12 (11.7)	0	12 (9.6)	
If yes, how did it change?				
Decided not to have the service	2 (1.9)	0	2 (1.6)	>.99
Decided to have service elsewhere (not at Sutter Health)	2 (1.9)	0	2 (1.6)	
Decided to have service at Sutter Health	4 (3.9)	0	4 (3.2)	
Other	2 (1.0)	2 (9.1)	4 (3.2)	
Contacted clinician				
No	97 (94.2)	21 (95.5)	118 (94.4)	>.99
Yes	6 (5.8)	1 (4.5)	7 (5.6)	

Abbreviations: GED, general education diploma.

^a^ Accuracy was defined as a difference between the estimate and billed amount of less than $10 or 5%.

In response to what patients liked about the cost estimator tool, 53 participants (42.4%) reported being able to anticipate costs (Table 3). One patient wrote, “It [the cost estimator tool]…gave a quick estimate of the cost of a service which I had never had before. It’s a relief knowing in advance how much you will be spending on healthcare.” Thirty-five respondents (28.0%) with accurate or inaccurate estimates responded that the tool was easy to use and had a straightforward and intuitive interface.

**Table 3.** Categories of Responses and Selected Examples From Open-Ended Survey Questions About Sutter Cost Estimator Tool

Response	No. (%)		
	Estimate Accurate^a^ (n = 103)	Estimate Not Accurate (n = 22)	Total (N = 125)
Likes			
Anticipate cost	44 (35.2)	9 (7.2)	53 (42.4)
Ease of use	26 (20.8)	9 (7.2)	35 (28.0)
Access	15 (12.0)	7 (5.6)	22 (17.6)
Accurate	5 (4.0)	3 (2.2)	8 (5.9)
Nothing, inaccurate, or not for all	8 (6.4)	0	8 (6.4)
Missing	9 (7.2)	2 (1.6)	11 (8.8)
Potential improvements			
More procedures and services	31 (24.8)	4 (3.2)	35 (28.0)
Nothing or NA	22 (17.6)	4 (3.2)	26 (20.8)
More accurate estimates	13 (10.4)	4 (3.2)	17 (13.6)
Information on deductibles and coinsurance	9 (7.2)	2 (1.6)	11 (8.8)
Technical capabilities	7 (5.6)	1 (0.8)	8 (6.4)
Accessibility	4 (3.2)	2 (1.6)	6 (4.8)
Clearer	4 (3.2)	1 (0.8)	5 (4.0)
Missing	16 (12.8)	3 (2.4)	19 (15.2)

Abbreviation: NA, not applicable.

^a^ Accuracy was defined as a difference between the estimate and billed amount of less than $10 or 5%.

The most frequently received improvement suggestion was to expand the procedures and services for estimates, given by 35 respondents (28.0%). However, 26 respondents (20.8%) expressed that no changes were needed because the tool worked as promised. One patient indicated that “At this time, I feel there are no improvements needed. The estimator gave me the approximate cost of my ultrasound which helped me in my financial planning.” Other suggestions for improvement included additional financial information that included coinsurance and deductibles.

## Discussion

This quality improvement study reported that the implementation of the patient cost estimator tool delivered more than 3500 online cost estimates in a 10-month period. For individuals with an EOB, the tool provided estimates with 83.9% accuracy. Initial survey results found that most respondents felt favorable about the overall experience, would use the tool again, would recommend it to others, and had an improved perception of their care at Sutter Health. A small minority of individuals contacted their clinician about estimates, suggesting that many consumers understand that their clinician does not have access to complex insurance information.^15^

While more than half of participants indicated the importance of knowing potential costs, few ultimately changed their decision because of the cost estimate. This is similar to the finding of a 2016 study^6^ that there was no association between price transparency tool use and outpatient cost savings. These findings suggest price transparency will not discourage patients from receiving health care services and that federal policies to increase price transparency alone may not be sufficient for delivering significant cost savings. However, while respondents did not report changing their health care decisions, they did report benefiting from the tool in fiscal planning. Thus, the overall value of the cost estimator to patients could be more difficult to measure, as they may be using other strategies, such as delaying care, placing money into a health savings account, or obtaining services elsewhere.

Developing a useful and accurate fee estimation tool is not trivial. Traditional fee estimation tools allow patients to query fee schedules but are not informed by the patient’s individualized health care benefit design. Despite Sutter Health collaborating with industry partners, our findings suggest that opportunities to further improve the quality of fee estimates exist, particularly with reduction of the variation of data sent by payers, access to a wider number of procedures, and improved timeliness of data updates to inform estimates.

### Limitations

This study has several limitations. This study included a small subset of the overall tool users (<10% of the total estimates created) who had an EOB. Of individuals invited to participate, a modest sample of tool users responded to the survey, and we do not know about the experience of nonrespondents or individuals who were not invited to complete the survey because we did not have an EOB for them. Additionally, participant responses are subject to recall bias because it may have been months since they used the cost estimator. The degree to which our experiences are generalizable to other health systems is not clear.

## Conclusions

While others have reported on the development and use of a cost estimator tool,^7,15,16^ this quality improvement study provided estimate accuracy rates and surveyed users to understand their experience with the tool. Nearly two-thirds of survey respondents noted the benefit of the estimator tool for fiscal planning, an important consideration as patients are increasingly sharing in the cost of health care. This reinforces the need and importance of delivering digital patient engagement solutions that support the holistic health care needs of the patient, inclusive of their clinical needs and the financial implications of care. Our experience provides an example of successfully implementing a cost estimator tool integrated with the online patient portal with a high degree of patient satisfaction. Other health systems may benefit from integrating a similar functionality.
